# Dermatofibrosarcome de Darier et Ferrand: à propos de 38 cas

**DOI:** 10.11604/pamj.2014.19.274.3604

**Published:** 2014-11-12

**Authors:** Hanan El Kacemi, Abdellah Aissa, Amine Bazine, Tayeb Kebdani, Abdeslam Bougtab, Noureddine Benjaafar

**Affiliations:** 1 Service de Radiothérapie, Institut National d'Oncologie de Rabat, CHU Ibn Sina, BP 6213 Rabat, Maroc; 2 Service de Chirurgie, Institut National d'Oncologie de Rabat, CHU Ibn Sina, BP 6213 Rabat, Maroc

**Keywords:** Dermatofibrosarcome, Darier et Ferrand, tumeurs cutanées, récidive, Dermatofibrosarcoma, Darier and Ferrand, cutaneous tumors, recurrence

## Abstract

Le dermatofibrosarcome protubérans de Darier et Ferrand est une tumeur cutanée rare. Le but de cette étude est de décrire les aspects épidémiologiques, cliniques, histologiques, thérapeutiques et évolutifs avec comparaison aux données de la littérature. Il s'agissait d'une étude rétrospective sur 10 ans à propos de 38 cas de dermatofibrosarcome de Darier et Ferrand pris en charge à l'Institut National d'Oncologie. Les données épidémiologiques, diagnostiques, thérapeutiques et évolutives ont été recueillies à partir des dossiers cliniques des patients et rapportées sur une fiche préétablie. L’âge médian des patients était de 41,5 ans. Tous les patients avaient une preuve histologique de dermatofibrosarcome. Seulement 30 patients ont été traités à l'Institut National d'Oncologie. L'exérèse chirurgicale de la tumeur était large, avec des marges de sécurité de 6-10 cm chez 6 patients (20%), 5 cm chez 16 patients (53%), 2-3 cm chez 4 patients (13%). En profondeur, l'exérèse emportait une barrière anatomique saine. 5 malades ont bénéficié d'une radiothérapie externe avec une dose médiane de 59,5 Gy. Le recul médian était de 64,4 mois (28- 138 mois). Le dermatofibrosarcome protubérans de Darier et Ferrand est une tumeur se distinguant par son évolution lente, son agressivité locale, son haut pouvoir de récidive et la rareté des métastases. Le traitement consiste en une exérèse large et profonde. Le pronostic dépend essentiellement de la qualité de l'exérèse initiale chirurgicale. La série étudiée présente des similitudes épidémiologiques, cliniques, et thérapeutiques avec les données de la littérature.

## Introduction

Le dermatofibrosarcome de Darier et Ferrand (DFS) est un sarcome des tissus mous de grade faible à intermédiaire provenant du derme de la peau. C'est une tumeur fibreuse cutanée rare représentant 0,1% des tumeurs cutanées malignes et 2 à 6% de tous les sarcomes du tissu mou. Le DFS touche préférentiellement les adultes jeunes entre 20 et 50 ans, il n'y a pas de prédominance nette concernant le sexe. Il se présente habituellement comme une masse cutanée nodulaire se localisant surtout au niveau du tronc et des extrémités. Son agressivité est avant tout locale, il n'existe pas de dissémination lymphatique et le taux de métastases générales est inférieur à 5%. La prise en charge thérapeutique fait appel à la chirurgie avec des marges larges de 3 à 5 cm pour réduire le taux de récidive, une exérèse marginale s'accompagne d'un taux de récidives locales de l'ordre de 40% [1, 2]. Son pronostic est lié à la qualité de son exérèse chirurgicale.

## Méthodes

Notre étude est une analyse rétrospective de 38 cas sur une période de 10 ans entre janvier 2000 et décembre 2009, incluant tous les patients porteurs d'un DFS recrutés à l'Institut National d'Oncologie (INO) de Rabat pendant cette période. Les données ont été extraites à partir du registre situé à l'unité d’épidémiologie de l'institut. Plusieurs paramètres ont été analysés notamment les données épidémiologiques, cliniques, anatomopathologiques, thérapeutiques et évolutives dans le but de proposer une meilleure conduite thérapeutique afin de réduire le risque de récidive et donc d'améliorer le pronostic.

## Résultats

L’âge concerne le moment où le diagnostic est posé, l’évolution de la tumeur étant lente, l’âge du début de l'apparition de la tumeur est difficile à établir avec précision. L’âge médian était de 41,5 ans avec des extrêmes de 9 ans à 85 ans. Les 38 patients se répartissent en 22 hommes (58%) et 16 femmes (42%), soit un sexe ratio de 3H/2F. Une notion de brûlure antérieure au site de développement de la lésion a été notée dans 2 cas soit 5,2% et la notion de traumatisme antérieur dans 1 cas soit 2,6%. Souvent les patients ont consulté suite à une croissance soudaine de la tumeur. Dans tous les cas, il existait une preuve histologique du diagnostic de DFSP. L'examen immunohistochimique à l'anticorps CD34 était réalisé dans 14 cas (47%) montrant une positivité avec un marquage CD34 cytoplasmique et membranaire. L’étude cytogénétique n'a pas été réalisée. Pour évaluer l'extension locorégionale, des radiographies standard ont été réalisées dans 34 cas, une échographie dans 20 cas et un scanner dans 3 cas. Le bilan d'extension à distance a consisté en une radiographie thoracique réalisée chez tous les patients. Elle était normale dans 34 cas, et montrait des lésions suspectes dans 4 cas où le bilan a été complété par un scanner thoracique. L’échographie hépatique a été Réalisée chez 22 malades et n'avait pas objectivé de lésions hépatiques suspectes. Seulement 30 patients ont été traités au sein de l'INO (79%) dont 12 de première main. Les autres patients ont été perdus de vue après leur première consultation et n'avaient reçu aucun traitement. Ces patients ont été exclus des résultats thérapeutiques. Chez les 30 patients opérés, L'exérèse chirurgicale de la tumeur était large, avec des marges de sécurité de 6-10 cm chez 6 patients (20%), 5 cm chez 16 patients (53%), 2-3 cm chez 4 patients présentant un DFS de l'extrémité céphalique (13%), non précisées chez 4 patients. En profondeur, l'exérèse emportait systématiquement une barrière anatomique saine. La réparation de la perte de substance engendrée par l'exérèse chirurgicale faisait appel aux différents procédés de la chirurgie plastique: Suture directe chez 23% des patients, une Cicatrisation dirigée dans 20% des cas, Greffe de peau réalisée immédiatement après l'exérèse chez 11 patients (43%). La couverture par lambeau était réalisée chez 6 patients (14%). 7 patients de notre série ont été repris chirurgicalement après analyse de la pièce d'exérèse qui a montré des limites atteintes. Les 23 autres cas présentaient des limites saines. Dans notre série, 5 malades ont bénéficié d'une radiothérapie externe, dans 2 cas pour des localisations empêchant une chirurgie large (localisation au niveau du sillon nasogénien et une localisation mandibulaire), dans 1 cas après une deuxième récidive, et dans 1 cas après des marges d'exérèse insuffisantes. La définition des volumes était basée sur l’évaluation préopératoire de la tumeur par l'examen clinique et les moyens iconographiques disponibles (scanographie, IRM). La dose médiane délivrée était de 59,5 Gy avec un fractionnement standard. L’étalement médian du traitement était de 34 jours. Deux patients ont été irradiés avec un accélérateur linéaire et trois patients avec un appareil de cobalt. Un seul patient a reçu une chimiothérapie adjuvante, il s'agissait d'un DFS transformé en un fibrosarcome avec dissémination au niveau pulmonaire. Le protocole était à base d'adriamycine-ifosfamide. Aucun de nos patients n'a reçu une thérapie ciblée. Tous nos patients ont été régulièrement suivis en consultation à des intervalles réguliers soit 1 consultation tous les 3 mois pendant les 2 premières années et tous les 6 mois par la suite. De principe on n'a pas fait de surveillance systématique par des examens complémentaires. Les résultats thérapeutiques sont exprimés dans notre série en terme de récidives, survenue de métastases et d'issu fatale, ils étaient appréciés avec un recul médian de 64,4 mois (28 mois à 138 mois). Ainsi, un cas de décès a été enregistré. Il s'agit d'un DFSP transformé en un fibrosarcome de grade II ayant disséminé au niveau pulmonaire. Le décès est survenu cinq mois après la découverte de métastase pulmonaire. Sur les 12 patients traités en première intention à l'INO, aucun n'a récidivé à ce jour. Sur les 20 patients traités en seconde intention dans notre formation, nous avons observé deux cas de récidives. Nous avons eu 3 perdus de vue dans cette série. Nous avons observé un seul cas de métastase pulmonaire survenu après 7 ans d’évolution d'un DFS.

## Discussion

Le DFS est une tumeur rare représentant 0,1% des tumeurs cutanées malignes. L'incidence mondiale est estimée entre 0,8 et 4,2 cas par million par an [3]. L’âge de survenue du DFS se situe généralement entre 20 et 50 ans. L’âge médian de nos patients était de 41,5 ans (9-85 ans). Le DFS est exceptionnel chez l'enfant et le nouveau né [4, 5]. Dans notre série, on note un seul cas de DFS de l'enfant. Le DFS intéresse les deux sexes avec une légère prédominance masculine [6]. Aucune relation de cause à effet n'a été démontrée; cependant, une notion de traumatisme antérieur est retrouvée dans 10 à 20% des cas [7]. Dans notre série, deux patients ont rapporté une notion de brûlure et un patient une notion de traumatisme. Sur le plan histopathogenique, l'origine histologique du DFS reste incertaine et discutée. Différentes origines sont évoquées par confrontation des données de l'immunohistochimie et de l'analyse en microscopie électronique: fibroblastique, histiocytaire, neuroectodermale [6]. L'aspect microscopique de la forme typique caractéristique est décrit pour la première fois par Taylor et Helwing en 1962 [7]. L'immunohistochimie est indispensable au diagnostic, ainsi le DFS exprime l'antigène CD34 et la vimentine [6, 8]. Dans notre série l'examen immunohistochimique à l'anticorps CD34 était réalisé dans 14 cas (47%) montrant une positivité de l'anticorps anti-CD34. Les techniques de cytogénétique mettent en évidence deux types d'anomalies du caryotype à type de chromosome en anneau surnuméraire r(17,22) ou une translocation t(17,22) [9]. La forme clinique classiquement décrite, protubérante, correspond à un stade avancé de la tumeur. C'est une masse ferme, multinodulaire, fixée à la peau en regard mais mobile par rapport aux plans sous jacents. La tumeur n'est pas douloureuse, sauf en cas d'ulcération [10]. La diversité des formes cliniques est une source du retard diagnostique. Le DFS peut survenir sur n'importe quelle partie du corps avec une prédominance au niveau du tronc et des extrémités [6]. Dans notre série, la localisation au tronc est la plus fréquente (67%). La chirurgie tient un rôle majeur dans le traitement curatif du DFS. Deux techniques d'exérèse permettent d'obtenir le contrôle tumoral dans plus de 90% des cas: l'exérèse large classique et la chirurgie micrographique de Mohs qui permet une exérèse tumorale avec réduction de la marge sous contrôle histologique de l'absence de cellules tumorales des berges d'exérèse [11–13]. L'exérèse classique consiste à effectuer nécessairement des excisions larges et profondes en emportant une marge périphérique en peau saine de 3 à 5 cm et en profondeur une barrière anatomique saine [5, 6, 14–19]. Il est évident que pour certaines localisations, telle que la face, cette marge de sécurité ne peut être respectée et devient même impossible. L'exérèse chirurgicale est alors réglée en fonction des notions de territoires anatomiques, fonctionnels et d'unités esthétiques [20]. Par ailleurs, lorsqu'il y a récidive, la marge d'exérèse doit être majorée. En ce qui concerne notre série, le protocole chirurgical a été homogène puisque plus de 70% des patients ont bénéficié d'une marge d'excision supérieure ou égale à 5cm, avec le sacrifice en profondeur d'une barrière anatomique saine. Seuls les DFS localisés au niveau du visage ou du cou n'obéissent pas à cette règle des 5 cm de sécurité, mais une marge de 3 cm a été respectée. Sur les 12 patients vus en première intention, aucun n'a récidivé, avec un recul médian de 64 mois (130 à 28 mois). Sur les 18 patients traités en deuxième intention, deux ont récidivé. Cette constations rejoint la littérature témoignant ainsi de l'importance du premier acte chirurgical. La reconstruction se fait par suture directe, cicatrisation dirigée ou par greffe cutanée ou lambeaux cutanés ou musculo-cutanés en fonction de la taille des lésions et leurs localisation. Le curage ganglionnaire systématique n'a aucun intérêt [7, 15, 21]. La plupart des auteurs rapportent le DFS comme une tumeur radiorésistante [7, 5, 22, 23]. Toutefois, d'autres ont affirmé que la radiothérapie diminuait le taux de récidives locales et permettait une chirurgie plus limitée [24–26]. Ainsi, selon HAAS et al, le control tumoral local était de 82% avec une radiothérapie adjuvante pour des marges d'exérèse insuffisantes ou envahies, avec un recul de 1 à 22 ans [25]. La radiothérapie est préconisée dans les récidives multiples, les marges d'exérèse insuffisantes ou envahies, les tumeurs de très grande taille et les localisations empêchant une chirurgie large [1]. L'association chirurgie radiothérapie semble avoir une efficacité sur la prévention des récidives [25–27]. La dose préconisée est de 50 Gray si chirurgie R0 et 60 Gray si chirurgie R1 répartis en 2 Gray par fraction, 5 jours par semaine [28]. Quant à la radiothérapie exclusive, elle peut être tentée dans les tumeurs inextirpables, en cas de patients inopérables ou refusant tout traitement chirurgical et la dose peut aller jusqu′à 66 Gray [1, 29]. Dans notre série, 5 patients ont bénéficie d'une radiothérapie externe et aucun d'eux n'a récidivé avec un recul médian de 5,3 ans. La Chimiothérapie est utilisée en palliatif en association avec la radiothérapie, mais elle ne semble pas être efficace. En effet, les multiples protocoles antinéoplasiques utilisant la doxorubicine, l'ifosfamide, le méthotrexate et la dacarbazine, n'ont montré aucune amélioration significative en terme de survie [14, 30]. Dans notre série, la chimiothérapie a été utilisée chez un seul patient présentant une métastase pulmonaire. La thérapie ciblée à base d'Imatinib Mesylate a été testée in vivo sur des tumeurs non résécables et dans des cas de DFS métastatiques. Son efficacité n'est pas totale et peut être nulle en l'absence de la translocation t(17,22) [31, 32]. Le pronostic du DFS est caractérisé par son fort potentiel de récidives. Le pourcentage de récidives varie en fonction des marges d'exérèses. L'exérèse chirurgicale initiale radicale est donc le facteur pronostique essentiel, conditionnant le risque de rechute locale. Quelques facteurs de mauvais pronostic ressortent des différentes séries publiées: l'exérèse incomplète, la localisation au niveau de l'extrémité céphalique, où les principes de l'exérèse large sont plus difficiles à respecter, l'existence de plages de fibrosarcome au sein de la tumeur, la profondeur de la tumeur [33, 34]. Le DFS ne métastase que rarement. Différentes séries retrouvent un taux de métastases de 3 à 5%, le pronostic de ces formes est sombre. La survie à 5 ans est estimée à 20% [35].

## Conclusion

Le DFS de Darier et Ferrand est une tumeur cutanée rare, à évolution locale lente s'effectuant sur plusieurs années. Elle se distingue par sa difficulté diagnostic, sa tendance à la récidive et la rareté de ses métastases qui sont essentiellement pulmonaires. Le diagnostic est souvent évoqué cliniquement et confirmé histologiquement, le recours à l'immunohistochimie se fait en cas de doute diagnostic (marquage de l'antigène CD34). Le traitement du DFS est chirurgical et doit répondre à un double objectif: pratiquer d'emblée une exérèse large passant entre 3 et 5 cm des berges selon la localisation et selon le caractère primaire ou récidivant de la tumeur, tout en s'efforçant d'emporter un plan sain en profondeur quel qu'il soit, et réaliser une couverture de la perte de substance engendrée par l'exérèse. Ce n'est qu’à ce prix que l'on peut espérer une guérison définitive. Une alternative au traitement chirurgicale classique est la résection selon la technique de MOHS qui permet d’économiser du tissu sain en se basant sur l'examen extemporané des berges de la pièce. Mais cette technique n'est pas encore pratiquée au Maroc en raison de son coût élevé et l'absence d’équipes entraînées. Ainsi, le DFS est de bon pronostic lorsque le traitement est bien mené mais exige néanmoins une surveillance clinique à vie sachant que certains cas de récidives ont été décrits très tardivement.
